# Wisconsin syndrome with brain volume laterality: a case report and review of the literature

**DOI:** 10.1186/s13256-022-03332-8

**Published:** 2022-04-16

**Authors:** Satomi Okano, Yoshio Makita, Kayano Kimura, Ikue Fukuda, Akie Miyamoto, Hajime Tanaka

**Affiliations:** 1 Department of Pediatrics, Hokkaido Asahikawa Habilitation Center for Children, 2-1-1-43 Shunkodai Asahikawa, Hokkaido, 071-8142 Japan; 2 Department of Genetic Counseling, Asahikawa Medical University Hospital, Hokkaido, Japan

**Keywords:** Wisconsin syndrome, 3q interstitial deletion, Brain volume laterality, *WWTR1*

## Abstract

**Background:**

Wisconsin syndrome is a congenital anomaly caused by a 3q interstitial deletion. It is associated with characteristic facies and developmental delays. Only 33 cases with a deletion estimated to be in the associated region 3q25 have been reported.

**Case report:**

We present the case of a 5-year-old Japanese girl with a 3q24q25.2 deletion. Her facial features corresponded to the Wisconsin syndrome phenotype, and she exhibited brain volume laterality, which has not been reported previously.

**Conclusion:**

The clinical features of our case may contribute to narrowing down the list of candidate genes of Wisconsin syndrome.

## Introduction

Wisconsin syndrome (WS) is a congenital anomaly caused by a rare 3q interstitial deletion. The region associated with WS is estimated to be 3q25. However, the causative gene remains unknown [1]. WS was proposed by Cohen and MacLean [2] on the basis of the clinical manifestations reported by Opitz in 1976. Ferraris *et al*. suggested that WS should be diagnosed when finding at least four of the five following core morphologic features: coarse face, prominent or wide triangular-shaped nasal tip, high arched or upsweeping eyebrows, full/everted lower lips, and bushy eyebrows, often with synophrys [3]. It is known that Dandy–Walker syndrome (DWS) is often a complication of WS. Here, we present the WS case of a Japanese girl with reduced volume of the right occipital lobe and thalamus, which had not been reported previously. Furthermore, we summarize the clinical features reported in the literature and discuss genes potentially responsible for WS.

## Case report

**Fig. 1.**
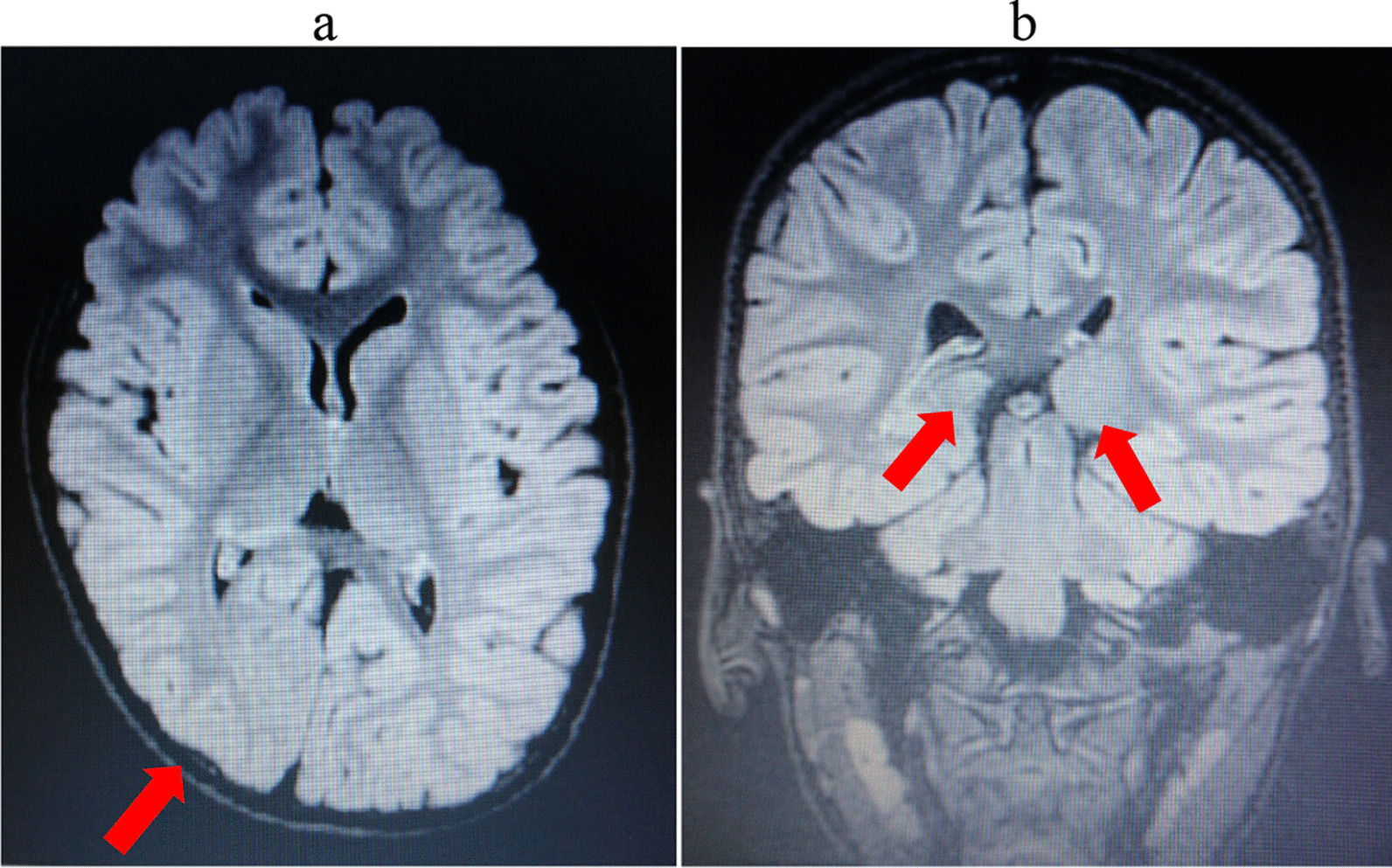
A T2-weighted magnetic resonance image of the brain showed (**a**) laterality of the right occipital lobe (red arrow) and (**b**) volume reduction of the right thalamus (red arrows show thalami)

The patient was the first girl born to healthy nonconsanguineous parents. She was born via normal transvaginal delivery at 40 weeks and 1 day of gestation, weighing 3340 g [± 0.85 standard deviation (SD)] with a length of 51.5 cm (± 1.47 SD) and an occipitofrontal circumference of 35 cm (± 1.50 SD). She could control her neck at 5 months, sit alone at 7 months, and walk independently at 16 months. Despite the absence of hearing loss, she could not speak meaningful words until 2 years of age. She was admitted to our hospital at 5 years of age. She had arched eyebrows, flat nasal tip, broad ala nasi, prominent ears, full everted lips, and a mouth that was always open. Cardiac auscultation results were normal. We did not find any abnormalities or laterality in the extremities. The deep tendon reflex was normal, and there was no paresthesia. She had mild intellectual disability (intelligence quotient 69 by the Tanaka–Binet intelligence test) and dysarthria, but she could read Japanese characters and count to 10. A vision test was not performed, and she went to kindergarten without trouble. There were no abnormal findings in the blood test. Brain magnetic resonance imaging revealed volume reduction in the right occipital lobe and thalamus (Fig. 1a, b). Circumference of right/left occipital lobe was 85.5 mm/88.4 mm, and that of right/left thalamus was 43.3 mm/68.6 mm. Laterality did not exist in the language center. Electroencephalography showed no laterality or paroxysm. She is currently receiving speech therapy at our hospital.

Ethical approval for this study was obtained from the Asahikawa Habilitation Center for Children Ethics Committee (ref. no. R01-19). After written informed consent was obtained from her parents in proxy of the patient, we performed a genetic examination. The results of the chromosomal analysis were 46, XX, del [3] (q25.q25.3). The microarray analysis confirmed arr[hg19] 3q24q25.2 (147,510.640-154,810,046) × 1, a 7.3 Mb heterozygous interstitial deletion in the long arm of chromosome 3.

## Discussion and conclusions

We diagnosed our patient with WS because the facial appearance fulfilled the clinical criteria proposed by Ferraris *et al*. [3]. These clinical manifestations are seen frequently in WS cases; hence, they have high diagnostic value (Table 1).

**Table 1:** The proportion of previously reported clinical manifestations

Clinical manifestation	Positive/mentioned	Proportion (%)
High arched eyebrow^a^	13/13	100
Developmental delay	32/32	100
Coarse face^a^	21/22	95.4
Wide nasal tip^a^	19/20	95.0
Full/everted lower lip^a^	16/17	94.1
Bushy eyebrow^a^	12/14	85.7
Dandy–Walker syndrome	17/21	81.0
Smooth philtrum	8/10	80.0
Ear anomalies	14/18	77.8
Macrostomia	6/9	66.7
Recessed fourth foot	5/13	38.5
Cardiac defect	5/14	35.7

Ratio of the symptomatic case to cases that mentioned the presence of a symptom. ^a^Essential clinical manifestations for diagnosis suggested by Ferraris *et al*. [3].

A literature search using PubMed confirmed 33 cases of 3q interstitial deletions in WS patients, including 3q25, which is considered the associated region of WS (Table 2) [1–22]. One mosaic case was excluded from the analysis. The main clinical features of WS, including a specific facial appearance, developmental delay of various levels, and some lower limb anomalies such as recessed fourth toe or clubfoot, were found in these cases.

**Table 2.** Clinical manifestations of our case and previous reports

References	Deletion	DWS	Coarse face	Wide nasal tip	High arched eyebrow	Full everted lips	Bushy eyebrow	Smooth philtrum	Macrostomia	Developmental delay	Ear anomaly	Recessed fourth foot	Cardiac defect	Other features
Okano	3q24q25.2	−	+	+	+	+	+	−	+	+	+	−	−	
Franceschini [4]	3q23q26	NM	+	+	+	+	+	NM	NM	+	NM	NM	−	Deafness
Martsolf^5^	3q23q25	NM	+	+	NM	−	+	NM	NM	+	+	NM	+	
Cohen [2]	3q23q25	NM	+	+	+	+	+	+	−	+	+	+	NM	
Al-Awadi [6]	3q23q25	NM	+	+	+	+	+	NM	NM	+	+	+	+	Clubfoot
Alvard [7]	3q23q25	NM	+	+	+	+	−	−	+	+	+	−	+	
Robin [8]	3q25.1q26.1	NM	+	+	NM	+	NM	NM	NM	+	+	NM	NM	
Chandler [9]	3q23q25	NM	+	+	NM	NM	NM	NM	NM	+	+	NM	NM	
Slavotinek [10]	3q25	NM	+	+	NM	+	NM	NM	NM	+	+	NM	NM	
Costa [11]	3q22.2q25.1	NM	+	+	NM	+	NM	+	NM	+	−	NM	NM	Hypospadias
Sudha [12]	3q25.1q25.3	+	+	+	+	NM	+	+	−	+	+	−	+	
Ko [13]	3q24q26.1	NM	+	+	+	+	+	NM	NM	+	+	+	−	
Grinberg [14]^a^		+	NM	NM	NM	NM	NM	NM	NM	+	NM	NM	NM	
Rea [15]	3q22.3q25.1	NM	−	−	NM	NM	NM	NM	NM	+	+	−	+	
Lim [16]	3q22.3q25.1	+	+	NM	NM	NM	NM	NM	NM	+	Nm	NM	NM	
Tohyama [17]	3q23q25.1	+	+	+	NM	+	NM	NM	NM	+	NM	NM	NM	
Weber [18]	3q23q25.1	+	+	NM	NM	NM	NM	NM	NM	+	NM	NM	NM	CAKUT
Willemsen [1]	3q24q25.33	−	+	+	+	+	+	+	+	+	−	−	−	
Willemsen [1]	3q25.1q25.3	NM	+	+	+	+	+	+	−	+	−	−	−	
Moortgat [19]	3q25.1q25.3	−	+	+	+	+	+	+	+	+	+	−	−	Alopecia
D’Amours [20]	3q25.1q25.3	+	NM	NM	NM	NM	NM	NM	NM	NM	NM	NM	NM	
Ferraris [3]	3q22.3q25.3	+	+	+	+	+	+	+	+	+	+	+	−	
Ferraris [3]	3q22q26.1	−	+	+	+	+	−	+	+	+	−	+	−	Epilepsy,MPSIII
Chang [21]	3q25.33	−	+	+	+	+	+	NM	NM	+	+	−	−	
Bertini [22]	3q24q25.2	+	+	+	+	+	+	−	+	+	+	−	−	

Listed in chronological order.

+ present, − absent

*NM* not mentioned in the article, *DWS* Dandy–Walker syndrome, *CAKUT* congenital anomalies of kidney and urinary tract, *MPS* mucopolysaccharidosis

^a^Grinberg *et al*. [14] reported ten cases of DWS and developmental delay carrying a 3q interstitial deletion. Their deletions were: 3q22.2q25.3, 3q22q26, 3q23q25.3, 3q23q23.31, 3q23q25.1, 3q24q25.1, 3q22.2q25, 3q25.1q25.3, 3q22.3q25.2, and 3q22.1q25.1. Other clinical features were unclear.

Regarding brain anomalies, only DWS was reported, and we could not find any other case with laterality of brain parenchyma. Neurological symptoms of our patient included mild intellectual disability and dysarthria; therefore, the clinical significance of brain laterality was unclear. Further analyses, such as functional magnetic resonance imaging, are needed to understand the relationship between clinical symptoms and imaging results.

The prognosis of WS depends on the occurrence of serious complications such as cardiac disease. Previously reported cardiac anomalies include ventricular heart septal defects, truncus arteriosus, and mitral and tricuspid prolapse. Willemsen *et al*. reported a 60-year-old female WS patient with typical facial features and intellectual disability, but without visceral disease, except for primary amenorrhea [1].

The *zinc-finger protein of cerebellum 1* (*ZIC1*, MIM *600470) and *zinc-finger protein of cerebellum 4* (*ZIC4*, MIM*608948) genes on 3q24 are known to cause DWS [23]. Although DWS is frequently observed in WS, this complication does not depend on deletion of the 3q24 region, as shown in Table 2. This difference was not caused by the resolution of G-banding because an array analysis was delineated in at least ten WS cases and the results were similar. Thus, *ZIC1* and *ZIC4* might not be essential for brain malformations in WS.

In the 3q24q25.2 region deleted in our case, there are five long intergenic noncoding RNAs, 12 genes for which published symbols are not available, and 10 identified genes: *SMARCA3* (MIM*603257), *TM4SF1* (MIM*191155), *WWTR1* (MIM*607397), *GYG1* (MIM*603942), *CPB1* (MIM*114852), *CPA3* (MIM*114851), *AGTR1* (MIM*106165), *MBNL1* (MIM*606516), *ZIC1*, and *ZIC4*. Bertini *et al*. proposed *MBNL1* as a candidate gene for WS [22]. We speculate that *WWTR1* on chromosome 3q25.1 might also be a candidate gene in consideration of its encoded protein function. *WWTR1* is a key downstream component of the Hippo-YAP/TAZ pathway that regulates organ size and tissue homeostasis. It promotes growth and many mitogenic hormones and growth factors acting through G-protein-coupled receptors [24]. The brain volume difference in our case raises the possibility that WS occurs owing to the absence of *WWTR1*.

In summary, we present a case of WS with brain parenchyma laterality. To elucidate the genetic and molecular aspects of WS, an accumulation of cases is necessary.

## Data Availability

Not applicable.

## References

[1] Willemsen MH, Leeuw N, Mercer C, *et al*. Further molecular and clinical delineation of the Wisconsin syndrome phenotype associated with interstitial 3q24q25. Am J Med Genet A. 2011;155A(1):106–12. 10.1002/ajmg.a.33715 21204216

[2] Cohen MMJ, MacLean RA. editors. Wisconsin Syndrome. Craniosynostosis: Diagnosis, Evaluation, and Management, 2nd ed. Oxford University Press, New York, 2000; pp. 432–3.

[3] Ferraris A, Bernardini L, Sabolic Avramovska VS, *et al*. Dandy–Walker malformation and Wisconsin syndrome: novel cases add further insight into the genotype–phenotype correlations of 3q23q25 deletions. Orphanet J Rare Dis. 2013;8:75. 10.1186/1750-1172-8-75 PMC3667004 23679990

[4] Franceschini P, Cirillo Silengo M, Davi G, *et al*. Interstitial deletion of the long arm chromosome 3 in a patient with mental retardation and congenital anomalies. Hum Genet. 1983;64(1):97. 10.1007/BF00289488 6873944

[5] Martsolf JT, Ray M. Interstitial deletion of the long arm of chromosome 3. Ann Genet. 1983;26(2):97–9. 6604494

[6] Al-Awadi SA, Naguib KK, Farag TI, *et al*. Complex translocation involving chromosomes Y,1, and 3 resulting in deletion of segment 3q23→q25. J Med Genet. 1986;23(1):91–2. 10.1136/jmg.23.1.91 PMC1049553 3950944

[7] Alvarado M, Bocian M, Walker AP. Interstitial deletion of the long arm of chromosome 3: case report, review, and definition of a phenotype. Am J Med Genet. 1987;27(4):781–6. 10.1002/ajmg.1320270406 3122568

[8] Robin NH, Magnusson M, McDonald-McGinn D, *et al*. De novo interstitial deletion of the long arm of chromosome 3:46, XX, del (3)(q25.1q26.1). Clin Genet. 1993;44(6):335–7. 10.1111/j.1399-0004.1993.tb03911.x 8131307

[9] Chandler KE, de Die-Smulders CE, Engelen JJ, *et al*. Severe feeding problems and congenital laryngostenosis in a patient with 3q23 deletion. Eur J Pediatr. 1997;156(8):636–8. 10.1007/s004310050681 9266197

[10] Slavotinek AM, Huson SM, Fitchett M. Interstitial deletion of band 3q25. J Med Genet. 1997;34(5):430–2. 10.1136/jmg.34.5.430 PMC1050955 9152845

[11] Costa T, Pashby R, Huggins M, *et al*. Deletion 3q in two patients with blepharophimosis-ptosis-epicanthus inversus syndrome (BPES). J Pediatr Ophthalmol Strabismus. 1998;35(5):271–6. 10.3928/0191-3913-19980901-06 9782438

[12] Sudha T, Dawson AJ, Prasad AN, *et al*. De novo interstitial long arm deletion of chromosome 3 with facial dysmorphism, Dandy–Walker variant malformation and hydrocephalus. Clin Dysmorphol. 2001;10(3):193–6. 10.1097/00019605-200107000-00008 11446413

[13] Ko WT, Lam WF, Lo F, *et al*. Wisconsin syndrome in a patient with interstitial deletion of the long arm of chromosome 3: further delineation of the phenotype. Am J Med Genet. 2003;120(3):413–7. 10.1002/ajmg.a.20149 12838565

[14] Grinberg I, Northrup H, Ardinger H, *et al*. Heterozygous deletion of the linked genes ZIC1 and ZIC4 is involved in Dandy–Walker malformation. Nat Genet. 2004;36(10):1053–5. 10.1038/ng1420 15338008

[15] Rea G, McCullough S, McNerlan S, *et al*. Delineation of a recognizable phenotype of interstitial deletion 3(q22.3q25.1) in a case with previously unreported truncus arteriosus. Eur J Med Genet. 2010;53(3):162–7. 10.1016/j.ejmg.2010.02.008 20215058

[16] Lim BC, Park WY, Seo EJ, *et al*. De novo interstitial deletion of 3q22.3-q25.2 encompassing FOXL2, ATR, ZIC1, and ZIC4 in a patient with blepharophimosis/ptosis/epicanthus inversus syndrome, Dandy–Walker malformation, and global developmental delay. J Child Neurol. 2011;26(5):615–8. 10.1177/0883073810384996 21471554

[17] Tohyama J, Kato M, Kawasaki S, *et al*. Dandy–Walker malformation associated with heterozygous ZIC1 and ZIC4 deletion: report of a new patient. Am J Med Genet A. 2011;155A(1):130–3. 10.1002/ajmg.a.33652 21204220

[18] Weber S, Landwehr C, Renkert M, *et al*. Mapping candidate regions and genes for congenital anomalies of the kidneys and urinary tract (CAKUT) by array-based comparative genomic hybridization. Nephrol Dial Transplant. 2011;26(1):136–43. 10.1093/ndt/gfq400 20605837

[19] Moortgat S, Verellen-Dumoulin C, Maystadt I, *et al*. Developmental delay and facial dysmorphism in a child with an 89 Mb de novo interstitial deletion of 3q25.1-q25.32: genotype–phenotype correlations of chromosome 3q25 deletion syndrome. Eur J Med Genet. 2011;54(2):177–80. 10.1016/j.ejmg.2010.11.011 21167329

[20] D’Amours G, Kibar Z, Mathonnet G, *et al*. Whole-genome array CGH identifies pathogenic copy number variations in fetuses with major malformations and a normal karyotype. Clin Genet. 2012;81(2):128–41. 10.1111/j.1399-0004.2011.01687.x 21496010

[21] Chang YT, Wang CH, Chou IC, *et al*. Case report of chromosome 3q25 deletion syndrome or mucopolysaccharidosis IIIB. Biomedicine. 2014;4(1):34–7. 10.7603/s40681-014-0007-0 PMC4264972 25520920

[22] Bertini V, Orsini A, Mazza R, *et al*. A 6.5 mb deletion at 3q24q25.2 narrows Wisconsin syndrome critical region to a 750kb interval: a potential role for MBNL1. Am J Med Genet A. 2017;173(1):280–4. 10.1002/ajmg.a.38002 27753286

[23] Blank MC, Grinberg I, Aryee E, *et al*. Multiple developmental programs are altered by loss of Zic1 and Zic4 to cause Dandy–Walker malformation cerebellar pathogenesis. Development. 2011;138(6):1207–16. 10.1242/dev.054114 PMC3042874 21307096

[24] Yu FX, Zhao B, Guan KL. Hippo pathway in organ size control, tissue homeostasis, and cancer. Cell. 2015;163(4):811–28. 10.1016/j.cell.2015.10.044 PMC4638384 26544935

